# Special Issue “Antibody Engineering for Cancer Immunotherapy”

**DOI:** 10.3390/antib11020029

**Published:** 2022-04-15

**Authors:** Silvia Crescioli, Ann L. White, Sophia N. Karagiannis

**Affiliations:** 1 St. John’s Institute of Dermatology, School of Basic & Medical Biosciences, King’s College London, Guy’s Hospital, London SE1 9RT, UK; sophia.karagiannis@kcl.ac.uk; 2 UCB Celltech, Slough SL1 3WE, UK; ann.white@ucb.com; 3 Guy’s Cancer Centre, Breast Cancer Now Research Unit, School of Cancer & Pharmaceutical Sciences, King’s College London, London SE1 9RT, UK

Since the approval of Rituximab in the late 1990s, the first chimeric monoclonal antibody for the treatment of non-Hodgkin lymphoma, antibody engineering for cancer immunotherapy has become a rapidly growing field, with almost 50 antibody therapeutics approved in the USA and EU and hundreds undergoing testing in clinical trials. Monoclonal antibody-based therapeutics have become a main component of cancer therapy, together with surgery, radiation, and chemotherapy. Novel research in the field of antibody engineering spans across many areas. These include antibody variable region humanization and optimization, Fc engineering, isotype optimization and antibody glycoengineering, as well as the design of complex antibody formats such as antibody-drug conjugates (ADCs), immunocytokines, antibody fragments, bi- and multispecific antibodies and cell engagers.

This Special Issue on “Antibody Engineering for Cancer Immunotherapy” is composed of two original and eight review articles covering various aspects of the growing field of antibody therapeutics for cancer treatment. Themes cover novel strategies for Fc engineering and glycoengineering, the use of alternative isotypes, such as IgE and IgA, ADCs, immuno-cytokines, multispecific antibodies and cell engagers. 

Monoclonal antibodies can have direct effects on tumor or immune cell functions and can also induce long-lasting anti-tumor immune responses. Zahavi and Weiner [1] provide an expert perspective of antibody immunotherapy, with a global overview of the known mechanisms of action, current clinical applications and mechanisms of resistance of monoclonal antibodies for the treatment of cancer. They further discuss combination therapies and how monoclonal antibody-based strategies have now moved towards targeting immune cells instead of tumor antigens as a mechanism of enhancing anti-tumor immune responses. 

With regards to the challenges in monoclonal antibody development, Charrin and colleagues [2] report a novel method for the rapid selection of rare isotype variants of the anti-CD63 TS63 antibody by sorting hybridoma cells on the basis of their high expression of surface immunoglobulins of the IgG2a and IgG2b subclass, followed by a limited dilution cloning step. This method can be readily applied for the selection of murine antibodies to be used in the preclinical steps of therapeutic antibody development for cancer immunotherapy. 

Antibodies are glycoproteins, decorated with glycans. The presence, absence and composition of glycans can have a profound effect on antibody pharmacodynamic and pharmacokinetic properties. Antibodies and their glycan composition have therefore gained a great interest from a technological, therapeutic, and regulatory perspective. Li and colleagues [3] review the main known effects of glycosylation on the biological and pharmacological functions of IgG, IgE, IgA, IgM and IgD isotypes. Furthermore, they provide examples for the use of small molecule inhibitors of glycan biosynthesis for application in antibody glycoengineering, discussing the advantages and challenges of this approach. 

Based on their Fc domain, antibodies can engage the C1q complement component or Fc receptors on immune cells, linking exquisite specificity for cancer antigens to powerful cellular and complement-mediated effector functions. Research dating back several decades has focused on elucidating the important role of glycosylation and has led to the identification of key amino acid residues crucial for Fc mediated functions. This has precipitated a growing interest in Fc-engineering to modulate antibody Fc effector function and antibody design approaches to influence specific mechanisms of action and therapeutic purposes. Liu and colleagues from the Beers and Cragg groups [4], leaders in antibody structure and functional links, provide a comprehensive review of the Fc domain properties crucial for antibody effector function (engagement of Fc receptors and complement) and behavior (such as engagement of neonatal Fc receptor). They discuss how the Fc domains can be engineered and glycoengineered to suit specific therapeutic purposes in the context of IgG class immunotherapy for cancer. 

All the monoclonal antibodies currently approved for clinical use are of the IgG isotype. IgA could offer a valid alternative to complement the therapeutic armamentarium which is currently composed of IgG class antibodies of different isotypes. The review by van Tetering and colleagues [5], from the Leusen group, a leading team in the study of IgA as a therapeutic antibody class, provides an overview of both the potential and the limitations of IgA antibodies as an alternative to IgG for cancer immunotherapy. When engaged by granulocytes, IgA antibodies targeted against cancer antigens are able to trigger superior tumor killing compared to IgG. Some properties of IgA, however, pose technical limitations such as challenges with production and purification, a heterogeneous glycosylation profile, and short serum half-life. The review focuses on the engineering strategies to overcome these technical limitations as well as alternative approaches using IgA/IgG hybrid and FcαR-engagers and discusses the impact of engineering on the planned clinical application of IgA for cancer immunotherapy. 

Another isotype which offers a valid alternative to IgG therapeutics is Immunoglobulin E (IgE). IgE antibodies are mainly known for their role in allergic diseases and antiparasitic immune responses. However, this isotype can mediate powerful effector functions which may be redirected for the treatment of cancer. Chauhan and colleagues [6], from the Karagiannis group, pioneer and leader for the use of IgE in cancer immunotherapy, provide a comprehensive overview of the properties of IgE. They describe the attributes which may enable IgE to engender superior antitumor activity compared to IgG class antibodies. This review focuses on the properties of anti-tumour IgE applied against cancer cells in vitro and in vivo, both with regards to efficacy and mechanisms of action. Given the perceived risk of type I hypersensitivity reactions which may be associated with IgE administration, the authors discuss safety considerations for the application of IgE as a therapeutic. They explore in vitro studies of potential hypersensitivity, the use of appropriate in vivo animal models for safety, possible implications of the high degree of glycosylation, characteristic of this isotype, and the use of ex vivo predictive and patient monitoring clinical tools. They further discuss the first-in-human clinical trial of a candidate anti-cancer IgE therapeutic antibody in terms of preliminary outcomes and the employed risk mitigation steps. 

Although effector functions are not perceived as key in the therapeutic functions and clinical application of ADCs, antibody engineering strategies can also be applied to modulate the effector function of ADCs. ADCs are composed of an antibody moiety to allow specific cell targeting, and a drug moiety responsible for the antibody’s cytotoxic effect. The field of ADCs is currently undergoing a significant expansion. Alongside the excitement in the field, we are seeing several strategies for antibody engineering designed to modulate the pharmacological properties of ADCs. Lucas and colleagues [7] provide a comprehensive overview of antibody engineering to alter the interaction of ADCs with the immune system, to influence pharmacokinetics, pharmacodynamics and the therapeutic index of ADCs. The authors highlight interesting engineering approaches which can be employed for the design of the next generation of ADCs.

The field of multispecific biotherapeutics, drugs able to engage two or more protein targets with or without chemical conjugation to large or small molecules, is rapidly evolving. These molecules can not only address specific intricacies of malignant diseases to be applied against stratified patient populations, but may also exploit new therapeutic mechanisms and access previously undruggable targets not available for use with conventional monospecific biologics. Zhong and D’Antona [8] provide an insightful review of recent advances in the molecular design, applications and challenges of the major classes of multispecific biotherapeutics, including immune cell engagers, ADCs, multispecific tetherbodies, biologic matchmakers, and small-scaffold multispecific modalities. Furthermore, the work by Cheng and colleagues [9] elucidates the molecular mechanism of the rapid HER2 internalization and degradation induced by a bispecific tetravalent anti-HER2 antibody (anti-HER2-Bs). This bispecific antibody is designed to bind two non-overlapping epitopes on the HER2 receptor, namely on domain IV (trastuzumab antibody epitope) and on domain II (39S antibody epitope). The authors report that while trastuzumab dissociated from HER2 in 2 hours, enabling the receptor to recycle, anti-HER2-Bs induce rapid internalization and degradation of HER2, whilst remaining associated with the receptor and promoting its ubiquitination, trafficking to the lysosomes and subsequent degradation. Their results enable a better understanding of the mechanism of action of anti-Her2-Bs and have the potential to guide the rational design of anti-HER2 therapeutics as well as of other bispecific molecules.

Another novel class of antibody-based therapeutics is immunocytokines. These antibody-cytokine fusion molecules aim to target cytokines directly into the tumour microenvironment. This class of agents has the potential to expand the therapeutic window of cytokine therapy by increasing efficacy and reducing toxicity compared to their cognate unconjugated cytokine. Runbeck and colleagues [10] provide a comprehensive review of the in vitro, in vivo, and clinical evidence for the application of immunocytokines in immuno-oncology.

The 10 articles featured in this Special Issue provide novel insights and information on various aspects of engineering of antibody therapeutics for cancer treatment, which are of considerable interest to many readers working on different aspects of cancer immunotherapy with antibodies. It is likely that the next generation of antibody therapeutics for cancer will arise from and be inspired by several aspects of the design approaches presented and discussed in the articles of this Special Issue.
